# When Light Drives Discovery: Plasmonics at the Frontier of Energy Materials

**DOI:** 10.1021/acs.chemmater.5c02994

**Published:** 2026-03-28

**Authors:** Khushboo Singh, Atif Jan, Giuliana Di Martino

**Affiliations:** Department of Materials Science and Metallurgy, 2152 University of Cambridge, 27 Charles Babbage Road, Cambridge CB3 0FS, U.K.

## Abstract

Plasmon-enhanced optical techniques provide unprecedented sensitivity for operando characterization of energy materials and devices. Yet their adoption remains limited, shaped by a combination of genuine technical challenges and perception-driven concerns: where issues such as experimental complexity, difficulties in signal interpretation, and perceived incompatibility with device operation collectively slow broader uptake. In this perspective, we critically examine the strengths and limitations of plasmonic optical methods, highlighting examples where they have delivered unique mechanistic insights. We discuss both technological and perceived challenges and outline strategies, such as standardized protocols, improved data analysis, and careful device integration, to broaden their use. By confronting misconceptions and practical hurdles, we argue that plasmon-enhanced techniques are poised to transition from niche tools to mainstream instruments for probing energy materials and devices under operational conditions, offering a path toward a deeper, real-time understanding of functional processes.

## Graphical abstract

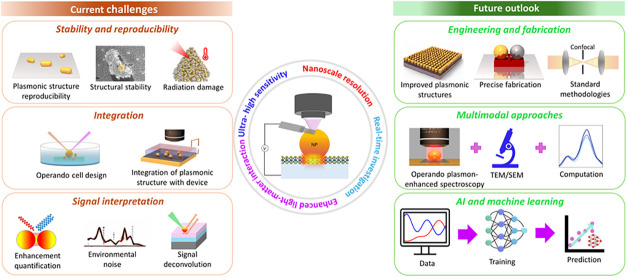

## 1 Introduction

Modern material science has reached a stage where advanced synthesis and nanofabrication techniques can produce highly engineered materials with atomic precision.^1,2^ Yet, translating these laboratory breakthroughs into practical technologies remains a formidable challenge. The key lies in understanding how these materials perform under realistic operating conditions.^3^ Observing their behavior during operation reveals the mechanisms that govern the performance, stability, and degradation. Such insights are vital not only for interpreting functionality but also for guiding the rational optimization of materials and devices.^4,5^ At this juncture, operando studies have become the cornerstone of material research. They provide the critical bridge between materials discovery and device application, enabling the transformation of promising concepts into reliable technologies.^6–8^ Its significance is evident from the surge in the number of publications and citations associated with the operando study of energy materials since 2014 (Figure 1 (top)).

**Figure 1:**
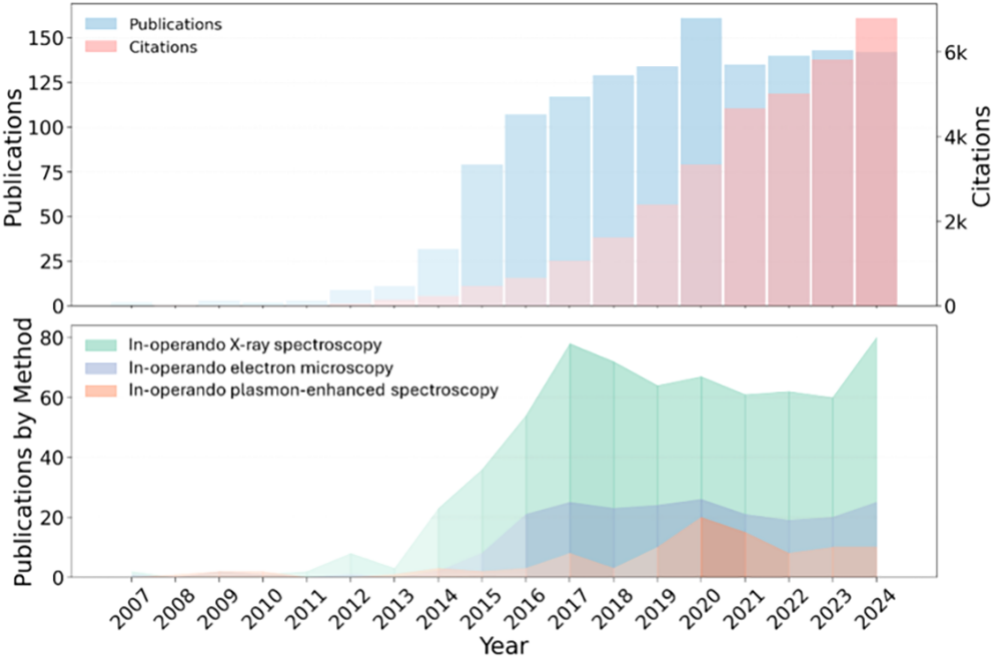
(Top) Number of publications and citations from 2007 to 2024 (keywords used: “in operando” and “energy materials”); (bottom) number of publications related to in operando studies of energy materials using different techniques.

Transmission electron microscopy (TEM), X-ray spectroscopy and scattering, and scanning probe microscopy (SPM) are among the well-established tools for in operando studies.^9–11^ They have unveiled nanoscale mechanisms that dictate the function and degradation of energy devices. These approaches have redefined how we understand materials in operation by providing direct quantitative access to structure–property relationships with an unprecedented resolution. However, when it comes to operando studies, these techniques fail to truly replicate the real operating conditions as they need ultrahigh vacuum and restricted sample specification. Other challenges are electron-beam damage and dependence on large-scale facilities (like a synchrotron).^9,12^ With the field on the brink of transformation, emerging noninvasive approaches are reshaping the balance between sensitivity, resolution, and practicality. Methods enabling nanoscale enhanced investigation offer a powerful platform for real-time investigation of device dynamics. By simultaneously amplifying and confining light–matter interactions at the nanoscale, these techniques deliver unprecedented spatial resolution (a few nanometers) and detection sensitivity.^13,14^ Additionally, their ability to operate under ambient conditions further enhances their suitability for operando studies. However, despite these advantages, their adoption in in operando studies remains limited (Figure 1 (bottom)) and reflects a coupled set of challenges rather than a single dominant barrier. Genuine technical difficulties, including the reproducible fabrication of plasmonic substrates, quantitative comparison of enhancement factors, and probe-induced perturbations, have slowed the establishment of standardized experimental protocols. In turn, the absence of such standards reinforces conservative methodological preferences and skepticism within the broader community. Recognizing this interplay between technical maturity and cultural adoption is essential for placing plasmon-enhanced approaches within a realistic and complementary operando toolbox.

In this perspective, we highlight the fundamental advantages and critical design considerations of these emerging techniques, emphasizing how the coupling of light with localized surface plasmon (LSP) resonances enables unprecedented sensitivity to ultrafine structural and electronic dynamics. We further explore their technological evolution and evaluate their capabilities through key pioneering studies, establishing performance benchmarks in spatial resolution, environmental adaptability, and data interpretability. Subsequently, we consider the major barriers impeding broader adoption, including the need for technical standardization, perception bias, and strengthened interdisciplinary integration. Finally, we outline potential directions for accelerating their development and translation into routine energy materials research.

## 2 Plasmon-Enhanced Optical Spectroscopy: Novel In Operando Characterization Tool

Illumination of metallic nanostructures with photons of specific wavelengths excite LSPs, that is, collective oscillations of conduction electrons confined to the nanostructure (Figure 2a).^15^ This electron–photon interaction generates intense electromagnetic fields at the nanoscale, often surpassing the diffraction limit. Highly localized regions of field enhancement, typically found at sharp features or in narrow interparticle gaps, are known as hotspots (Figure 2b). Materials, specifically the atomic bonds present within the nanoscale hotspot experience enhanced optical fields (∼*E* ^4^), leading to the enhanced processes such as Raman scattering, fluorescence, or absorption.^16,17^ The properties of LSPs are strongly influenced by the nanostructure’s composition, size, shape, and local dielectric environment, offering a sensitive probe of nanoscale structural and chemical variations.^18^

**Figure 2:**
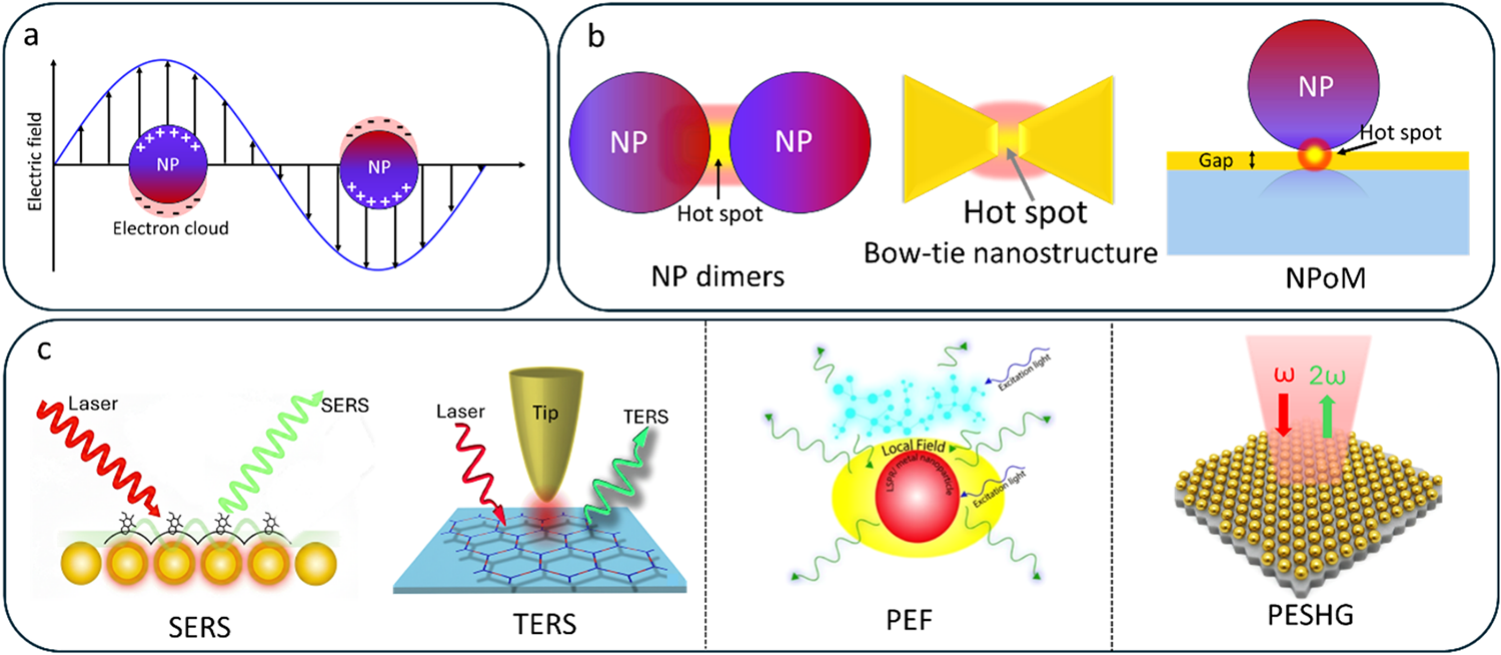
(a) Schematic showing localized surface plasmon resonance (LSPR) in metal nanoparticle (NP); (b) schematic representation of typical geometries used to achieve plasmonic enhancement; and (c) some of the key plasmon-enhanced techniques. NPoM: nanoparticle on mirror, SERS: surface-enhanced Raman spectroscopy, PESHG: plasmon-enhanced second harmonic generation, PEF: plasmon-enhanced fluorescence, PESHG: plasmon-enhanced second harmonic generation, TERS: tip-enhanced Raman spectroscopy.

Localized surface plasmon resonances (LSPRs) significantly enhance a wide range of light–matter interactions, including fluorescence, Raman scattering, photothermal and photoacoustic effects, photocatalysis, and nonlinear optical conversion.^19^ Over the past four decades, this phenomenon has driven the development of a wide range of plasmon-enhanced spectroscopic techniques (Figure 2c), including surface-enhanced Raman spectroscopy (SERS), surface-enhanced infrared absorption (SEIRA), plasmon-enhanced fluorescence (PEF), and tip-enhanced Raman spectroscopy (TERS).^20^ Plasmon-enhanced spectroscopy can be realized through diverse nanostructural configurations offering distinct advantages in field enhancement, spatial confinement, and experimental versatility. While these techniques have long been applied in ex situ chemical and structural analyses, recent advances in nanofabrication and optical engineering have expanded their use in in operando studies, opening new opportunities for real-time probing of energy materials at the nanoscale.^21–24^

Plasmon-enhanced techniques have recently been applied to investigate batteries under real operating conditions, offering powerful optical sensitivity to interfacial and structural changes. A notable example is the work of Miroshnikov et al. that employed Raman spectroscopy enhanced by plasmonic nanoparticles (Au and Ag) in silicon anode materials for Li-ion battery.^25,26^ They demonstrated that the plasmonic nanoparticle decoration enabled surface-enhanced Raman signals in a working cell environment, thereby providing improved sensitivity to phase changes and electrode behavior during cycling, otherwise inaccessible to conventional Raman (Figure 3a). Another striking example is the work by Jin et al. that utilized plasmonic reflectance spectroscopy to monitor lithium–metal deposition under operando conditions.^27^ The work monitors lithium–metal deposition processes, where the evolution of plasmonic reflection dips directly correlates with the nature of Li growth. It shows that ordered nanoparticle deposition produces sharp, size-dependent resonances, while dendritic or irregular growth yields broad or suppressed responses (Figure 3b). Beyond providing a powerful diagnostic of lithium dynamics, this work exemplifies how plasmonic spectroscopy can deliver insights inaccessible to conventional X-ray or electron microscopy, offering a complementary route to probing reactive and beam-sensitive electrochemical interfaces. A recent study by Laurenti et al. extended the applicability of the plasmon-enhanced technique by employing TERS to track nanoscale Li-ion dynamics in a working battery electrode. The work investigates model cathode thin films to resolve spatial ion dynamics and time-resolved phase transition at the nanoscale.^28^ The TERS time map (Figure 3c) shows the enhancement of the peak associated with λ-MnO_2_ phase with the positive current peak of cyclic voltammetry and, hence, demonstrates the ability of operando TERS to map phase transition with nanoscale resolution.

**Figure 3:**
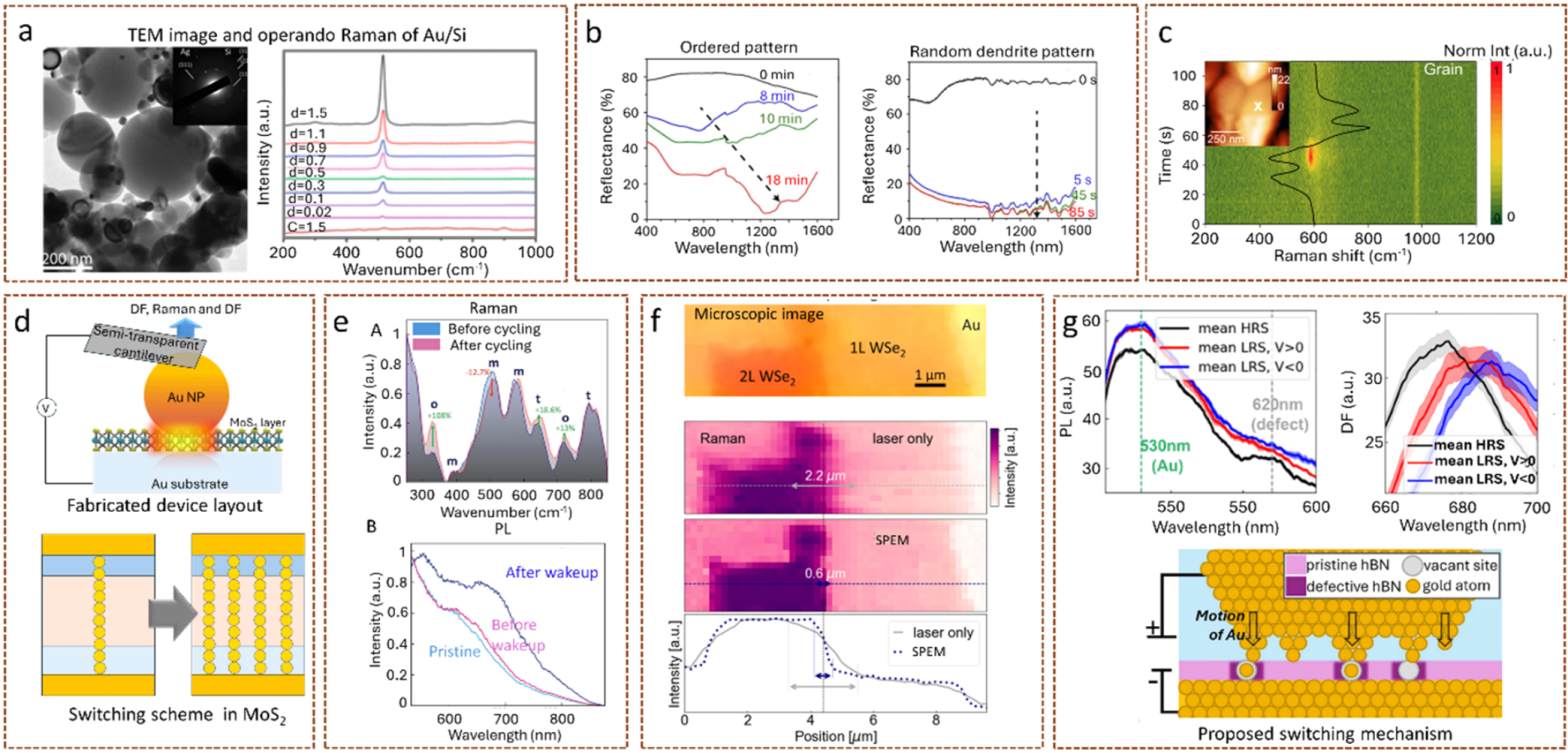
(a) Transmission electron microscopy (TEM) images and operando SERS showing lithiation–delithiation in Au/Si anode [adapted with permission from^26^; copyright 2018 American Chemical Society]; (b) in operando reflectance spectroscopy showing different pathways of lithium evolution [adapted with permission from^27^ copyright 2018 Proceedings of the National Academy of Sciences]; (c) temporal and spatial map of TERS showing ion dynamics in LiMn_2_O_4_ model cathode film [adapted with permission from^28^ copyright 2025 Royal Society of Chemistry]; (d) operando plasmon-enhanced optical studies in MoS_2_ memristors [adapted with permission from^30^; available under CC-BY 4.0; copyright 2022 Wiley]; (e) Raman and photoluminescence (PL) in AuNP/HZO/TiN ferroelectric memory devices to study wake-up and fatigue [adapted with permission from^31^ available under CC-BY 4.0; copyright 2023 Wiley]; (f) microscopic image and Raman maps, with SPEM improving spectral resolution from ∼2.2 μm to ∼600 nm [adapted with permission from^32^; available under CC-BY 4.0; copyright 2024 American Chemical Society]; (g) PL and dark-field (DF) spectra of hexagonal boron nitride (hBN) memristors and proposed switching mechanism, HRS: high resistance state and LRS: low resistance state [adapted with permission from^33^; available under CC-BY 4.0; copyright 2025 Wiley].

Beyond electrochemical energy systems, plasmonic techniques have also been pioneeringly employed to study emerging nanoscale devices under operation.^29^ For instance, Symonowicz et al. employed plasmon-enhanced optical spectroscopy to investigate switching mechanism in MoS_2_ in ambient condition with nanoscale resolution.^30^ The authors fabricated cells with 1–3 layers of MoS_2_ and probed excitonic and defect-related modes, tracking otherwise weak vibrational features. The operando PL, Raman, and DF, supported by finite difference time domain (FDTD) simulation, reveal that the switching is a result of Au intercalation into MoS_2_, not mediated by defect creation, while highlighting interface engineering as the main potential route to tailor MoS_2_ memristive behavior (Figure 3d). Similarly, Jan et al. employed plasmon-enhanced in operando spectroscopy to probe 5 nm ferroelectric Hf_0.5_Zr_0.5_O_2_ (HZO) films, enabling the first real-time optical tracking of oxygen vacancy migration and phase transitions in ultrathin HZO films.^31^ Using a NPoM geometry, simultaneous Raman and PL measurements captured vacancy dynamics and structural evolution with single switching cycle resolution, directly linking these processes to wake-up and fatigue behavior in ferroelectric memories (Figure 3e). The operando SERS spectra reveal that after cycling, there is a significant increase in the o/t (orthorhombic/tetragonal) phase and a decrease in the m (monoclinic) phase. The PL result confirmed the role of oxygen vacancy in the phase transition.

Complementing this development, Symonowicz et al. introduced scanning plasmon-enhanced microscopy (SPEM), which integrates the NPoM geometry with a portable conductive cantilever to enable simultaneous optical, electrical, and topographical measurements.^32^ Applied to MoS_2_ and WSe_2_ nanosheets, SPEM achieved ∼600 nm spatial resolution and high optical enhancement while forming pristine, nondestructive van der Waals electrical contacts (Figure 3f). The actual spatial resolution can be much smaller (tens of nanometers) determined by the size of NP facet. More recently, Kelly et al. employed plasmon-enhanced optical spectroscopy to reveal point defect-mediated conductive filament formation that switches the device. The PL spectra corresponding to HRS show an additional peak at 620 nm, which vanishes at the LRS state, indicating that the Au atom modifies the defect state, and voltage-induced local refractive index modifications indicated by DF spectra support the PL results (Figure 3g). Hence, this work highlights the critical role of defects in hBN switching dynamics and underscores defect engineering as a strategy to optimize device performance.^33^

## 3 Understanding the Gap: Barriers to Adoption

A key concern remains the limited adoption of these nonconventional techniques as reflected by their modest publication volume. This challenge can be addressed through a two fold strategy: first, by identifying the barriers, and second, by developing strategies to integrate these methodologies into the mainstream operando characterization workflow. From our perspective, there exist mainly two barriers: technological limitations and perception bias. Several review articles and perspectives have discussed the technological gaps in this pathway.^34,35^ Technologically, progress is often hindered by difficulties in the reproducible fabrication of plasmonic nanostructures with well-controlled geometry and subnanometre precision.^36^ Even slight variations in nanoparticle morphology, surface chemistry, or spacer thickness can lead to substantial variations in field confinement, resonance wavelength, and enhancement factors, thereby complicating comparison across research groups.^37^

Moreover, integrating these nanostructures into an active energy device further amplifies these challenges, as it demands specialized cell architectures that maintain optical access, mechanical robustness, and alignment throughout electrochemical cycling.^38^ Additionally, the local field enhancement may induce unwanted photothermal or electrochemical effects, particularly with high-power lasers, complicating the data interpretation. Plasmonic heating is an intrinsic consequence of LSPR which can result in steady-state temperature rise at nanoparticle surface as well as in surrounding medium within the hotspot.^39^ This local heating can modify the morphology of the plasmonic nanoparticles, introduce unintended changes in the sample, and even damage the sample. Moreover, these thermal perturbations can distort the true operando state of the device, limit measurement fidelity, and constrain the range of conditions under which reliable characterization can be performed. Consequently, these undesired effects, coupled with environmental fluctuations, introduce significant spectral variability, obscuring material information.

The factors outlined above collectively foster a perception that plasmon-enhanced operando techniques are difficult to implement and are still far from practical realization, which in turn slows their integration into mainstream materials and device research. High-impact journals and widely used research platforms tend to prioritize characterization methods that combine established performance metrics with broad community familiarity, particularly when quantitative interpretation is central. Because publication in these venues strongly influences scientific credibility, community confidence, and industrial engagement, emerging approaches are evaluated against stringent expectations. The comparatively limited presence of plasmonic operando studies therefore arises from the combined influence of remaining technical challenges and cautious methodological adoption rather than any single constraint. This interplay provides clear motivation for the outlook that follows, which outlines pathways through engineering, standardization, data intelligence, and dissemination to accelerate broader integration.

## 4 Outlook: Towards Mainstream Adoption

The broader adoption of operando plasmonic techniques in energy materials research requires coordinated progress across engineering, methodology, data interpretation, and community engagement. Rather than a single limiting factor, the current gap reflects a sequence of interdependent barriers that must be addressed systematically. A primary requirement for wider adoption is the development of reproducible and quantitatively reliable plasmonic platforms.

Progress in deterministic nanofabrication, including lithography-assisted assembly and atomic-layer-deposition-defined spacers, has begun to reduce the variability in plasmonic enhancement. Recent approaches such as nanoindentation-based lithography provide a promising pathway to directly pattern nanostructures with precise control over feature size and shape, yielding localized surface plasmon resonances tunable across the visible spectrum with good uniformity.^40^ Emphasis must shift from achieving maximum enhancement toward engineering a predictable and stable field confinement. Emerging architectures, such as NP/two-dimensional (2D) material hybrids and multifunctional heterostructures, are expected to redefine the operational landscape, offering superior stability, field enhancement, and spectral selectivity under working device conditions.^41,42^

Integrating plasmonic architectures directly into device-relevant geometries rather than relying on post hoc NP decoration will further improve mechanical stability and compatibility with operando environments. Beyond fabrication, methodological standardization is essential for establishing confidence in operando plasmonic measurements. Rather than relying on a single universal standard, standardized classes of probes and calibration platforms tailored to specific operando environments should be adopted. For instance, use of gas diffuse electrode cell incorporating SERS/SEIRA-active nanostructures for operando electrocatalytic study can be standardized as it operates close to industrially relevant condition.^14^ On the similar lines, since the devices under investigation are heterostructures, a confocal arrangement using a microscope objective is recommended, as it can deconvolute the contribution of individual layers from observed response.

Moreover, transparent reporting of core experimental parameters such as excitation conditions, power density, and acquisition protocols is also critical for a reliable operando plasmonic study. Importantly, these protocols must explicitly incorporate controls for probe-induced perturbations, including plasmonic heating, through power-dependent and time-resolved measurements. Furthermore, AI and machine learning (ML) can transform plasmon-enhanced operando studies from bespoke measurements into predictive, high-throughput platforms. In plasmonic nanostructure engineering, neural networks have demonstrated inverse design of metasurface geometries directly from optical spectra, enabling rapid fabrication of antennas tailored to maximize near-field enhancement at target vibrational bands, a capability critical for operando SEIRA/SERS sensitivity.^43^

More broadly, ML frameworks have predicted optical responses and mapped synthesis–structure–performance relationships of nano plasmonic materials, accelerating optimization of catalytic and sensing architectures without exhaustive parameter sweeps.^44^ For operando spectroscopy, supervised and deep-learning classifiers can quantitatively resolve overlapping and noisy specimen fingerprints, enabling a real-time investigation of device or system dynamics. Together, these capabilities establish closed-loop workflows including design, fabricate, measure, and retrain that reduce experimental burden and enable adaptive, data-guided plasmonic probes.

Even as technical and methodological maturity advances, deliberate dissemination will be essential to overcome perception-based barriers. Dedicated symposia and conference sessions, special issues and invited perspectives in high-impact journals, and tutorial-style best-practice articles can position plasmon-enhanced operando techniques alongside established characterization methods. With standardized practices, intelligent design, and open benchmarks, plasmon-enhanced operando spectroscopy is poised to evolve from a specialized demonstration into a robust, broadly deployable analytical platform for real-world devices.
